# An Agar–Water-Assisted OD_650_ Calibration Model for Standardized Quantification of *Beauveria bassiana* Conidia in Biopesticide Quality Control and Bioassay Applications

**DOI:** 10.3390/jof12060396

**Published:** 2026-05-29

**Authors:** Jie Cheng, Zhaoan Shao, Zhenxia Zhu, Shuohan Wang, Donghui Gong, Chengshuai Xu, Chaobo Zhang, Xiang Xiu, Yongcheng Ding

**Affiliations:** 1 State Key Laboratory of Macromolecular Drugs and Large-Scale Preparation, School of Pharmaceutical Sciences and Food Engineering, Liaocheng University, Liaocheng 252000, China; chengjie@lcu.edu.cn (J.C.); 18754003991@163.com (Z.S.); 17661802272@163.com (Z.Z.); 17308101168@163.com (S.W.); xiuxiang@lcu.edu.cn (X.X.); 2 School of Life Science and Technology, Inner Mongolia University of Science and Technology, Baotou 014010, China; gongdh1976@163.com; 3 Sino-Danish College, University of Chinese Academy of Sciences, Beijing 101408, China; chengshuaixu9926@126.com; 4 Shanghai Academy of Environmental Sciences, Shanghai 200233, China

**Keywords:** optical density, fungal propagules, calibration curve, suspension stability, microbial biopesticide, quality assessment

## Abstract

*Beauveria bassiana* is one of the most widely used entomopathogenic fungi in insect pest management, and the need for rapid and reproducible quantification of fungal conidia to monitor process performance and to quality control products during biopesticide production is imperative. Conventional methodologies, such as hemocytometer counting and plate dilution assays, are time consuming, laborious and subject to significant operator-to-operator variability. Although optical methods have been increasingly explored for estimating fungal propagule concentrations, species-specific calibration, suspension stability, wavelength selection, and independent validation remain important for routine applications. In this study, we developed an agar–water-assisted UV–visible spectrophotometric calibration protocol for estimating conidial concentration using *B. bassiana* as a model entomopathogenic fungus. A 0.1% (*w*/*v*) agar–water suspension was used in order to get homogeneous, stable dispersions of conidia for optical measurements. Calibration of conidia concentration was accomplished through reliable optical density (OD) values measured at wavelengths 500 nm, 530 nm, 560 nm, 600 nm, and 650 nm. Linear correlations were observed across the tested wavelengths, with the highest goodness of fit for the model at 650 nm (R^2^ = 0.9907). The resulting regression equation, conidia concentration (×10^7^ mL^−1^) = 4.184 × OD_650_—0.12450, has been independently verified with separate conidia batches, resulting in acceptable relative errors ranging from 13.78% and 18.98%. This agar–water-assisted OD_650_ calibration model provides a practical and species-specific tool for the standardization of conidial dosages in biopesticide research, facilitating the reliable evaluation and application of entomopathogenic fungi within integrated pest management systems.

## 1. Introduction

Entomopathogenic fungi play an important role in sustainable pest management due to their ability to infect and suppress a wide range of insect pests while reducing reliance on synthetic chemical pesticides [1,2]. As one of the most widely studied and commercially produced entomopathogenic fungi, *Beauveria bassiana* has been used in different cropping systems, including grains, cotton, vegetables, and horticultural crops [3,4]. In recent years, the global market of microbial biopesticides has significantly expanded due to increasingly strict regulations on synthetic pesticides and consumers’ growing preference for environmentally sustainable alternatives. The infectious propagules of *B. bassiana*, particularly aerial conidia, are the main active units in mycoinsecticide production and application, because they initiate host infection after contact with susceptible insects and are commonly incorporated into dry or liquid formulations for pest management [5,6,7]. Therefore, accurate and reproducible quantification of conidial concentration is essential for strain evaluation, fermentation monitoring, product standardization, dosage preparation, and bioassay reproducibility. However, several challenges still exist in the mass production and commercial promotion of biocontrol agents, such as poor batch-to-batch stability, inadequate formulation shelf-life, and a lack of standardized quality control systems for the determination of conidia concentration and viability [8,9].

In agricultural practice, the performance of microbial biopesticides is highly sensitive to variations in propagule concentration, which directly affect application dosage, infection pressure, and ultimately pest control efficacy. Conidia, which function as both reproductive and infectious agents of the entomopathogenic fungus, are critical components of host infection and distribution throughout the environment. Inconsistent quantification of conidia can lead to substantial variability among experiments and field applications, complicating the interpretation of biological control outcomes and limiting comparability across studies. For both basic research and practical applications, precise measurements of conidial density are required to ensure the development of comparable and reproducible datasets, such as those used in biological control, fermentation optimization and virulence testing [10,11]. In both industrial and regulatory environments, accurate quantification of the number of conidia in a sample is necessary for product standardization and to evaluate the efficacy of microbial plant protection products [12]. Consequently, the development of a simple and reproducible method for conidial quantification is not only a technical requirement but also a prerequisite for the standardization and reliable deployment of entomopathogenic fungi in agricultural pest management.

Conventional methods for determining fungal conidial concentration mainly rely on hemocytometer counting and plate dilution assays. Although these methods are inexpensive and relatively simple to use conceptually, they are highly labor intensive, require large amounts of time, and are highly dependent on operator skill, resulting in significant variation in measurement results [11]. Manual counting may introduce considerable variation among analysts, whereas plate dilution assays require incubation time and estimate only viable propagules under the selected culture conditions. These limitations become more evident when large numbers of samples must be analyzed during fermentation optimization, formulation development, routine quality control, or standardized virulence assays. Accordingly, rapid and reproducible alternatives are needed to support more efficient quantification of fungal conidia in both laboratory and applied biopesticide contexts.

To date, various alternative quantitative techniques have been developed. One of the most well-established methods used to quantify the number of cells in a suspension is UV–visible spectrophotometry due to its simplicity, speed, and reliable results. Recently, this method has also been adapted to fungal studies, and has been shown to be an effective measure of the concentration of conidia within multiple fungal genera, including *Fusarium* [13], *Penicillium* [14], *Trichoderma* [15,16], and *Cladosporium* [17], demonstrating that optical density is robustly, linearly related to conidia concentration within a specified range. In addition, several novel technologies have provided the possibility for high-throughput analysis of fungal conidia, which can further facilitate viability and physiological status analysis of conidia. For example, flow cytometry enables rapid, high-throughput detection and viability assessment of fungal propagules [18,19]. However, this method requires high equipment, fluorescent dyes, and skilled technicians, limiting its implementation for large-scale fermentation monitoring. Microscopy-based image analysis and machine learning tools can automate conidia counting at low cost under controlled imaging conditions; however, their performance often diminishes when evaluating complex mixtures in fermentation broths or formulated products, such as agglomerates, debris, or dissimilar backgrounds [20,21].

Among all the methods mentioned above, UV–visible spectrophotometry seems to be one of the most promising strategies for accurately measuring conidia concentration. However, it should be pointed out that fungal conidia exhibit significant differences in size, shape, color, and surface hydrophobicity, which directly affect their light scattering and absorption properties. Prior research has shown that morphological variations can have a significant effect on spectrophotometric measurement performance, and therefore specific calibration models need to be established for different species [16,17]. In addition, rapid settling characteristics for conidia in low-viscosity water-based suspensions can generate unstable signals during the process of spectrophotometric detection analysis, resulting in poor quantitative accuracy.

From a physicochemical point of view, increasing the apparent viscosity or adding weak structural networks to the suspension medium can reduce the rate at which particles settle and increase short-term dispersion stability, which will ultimately improve the reproducibility of measurements [22]. In addition to providing stability of the suspension, the choice of a particular wavelength can greatly affect the spectrophotometric sensitivity toward the suspension. Previous studies have demonstrated that the pigments produced by fungi as well as the cell wall components of fungal species have a significant effect on the absorption and scattering of light in the visible spectrum, resulting in different optical density measurements for different fungal species. The use of wavelengths in the vicinity of 530 nm has been widely adopted as a standard for the standardization of fungal inocula and has been reported to improve sensitivity compared to longer wavelengths, and the specific effect depends on the size and pigmentation of the reproductive body [23,24,25].

Since *B. bassiana* is an extensively studied entomopathogenic fungus that has been successfully applied in biological control, accurate quantification of its conidia is of great significance in strain screening, fermentation monitoring, formulation development, and standardized biological assays. Recent work has further extended optical quantification approaches to entomopathogenic fungi. Mora-Pérez et al. evaluated spectrophotometric and L*a*b* colorimetric methods for estimating spore concentrations in eight entomopathogenic fungi, including *B. bassiana*, and demonstrated that both optical approaches can be used for spore concentration estimation under defined analytical conditions [26]. Their study also highlighted the practical advantages of colorimetry over spectrophotometry in terms of a broader quantification range, especially for production and application scenarios [26]. These findings provide an important basis for optical estimation of entomopathogenic fungal spores and indicate that spectrophotometric quantification of *B. bassiana* has already been explored. Nevertheless, for routine *B. bassiana* quality control and bioassay applications, further development of a species-specific protocol emphasizing suspension stability, wavelength selection, working-range definition, and independent validation remains useful. Consequently, the objective of this study was to develop and validate a practical, species-specific OD-based spectrophotometric calibration protocol for rapid and standardized quantification of *B. bassiana* conidia in biopesticide quality control and bioassay applications.

## 2. Materials and Methods

### 2.1. Fungal Strain and Isolation

The fungal strain used in this study was isolated from *Bombyx mori* larvae infected with white muscardine disease (commonly known as “Baijiangcan”). Infected larvae were surface-cleaned, homogenized in sterile distilled water, and aliquots of the suspension were spread onto potato–potato dextrose agar (PPDA) plates. The plates were incubated at 27 °C for 5 days, after which single colonies with typical *Beauveria* morphology were selected and subcultured to obtain a pure isolate.

### 2.2. Culture Media and Cultivation Conditions

The PPDA medium was prepared in the laboratory rather than purchased commercially. Briefly, 200 g of peeled potatoes were boiled in distilled water, and the extract was filtered and adjusted to a final volume of 1 L. The medium was supplemented with glucose at 20 g/L and agar at 15 g/L, corresponding to final concentrations of 2.0% and 1.5% (*w*/*v*), respectively. The medium was sterilized at 121 °C for 20 min before use. For liquid culture, the basal medium comprised glucose (20.0 g/L), NaNO_3_ (2.0 g/L), yeast extract (2.0 g/L), KH_2_PO_4_ (1.0 g/L), MgSO_4_ (0.5 g/L), and NaCl (0.5 g/L), and was similarly sterilized at 121 °C for 20 min prior to use. All chemicals and reagents used in this study were purchased from Shanghai Aladdin Biochemical Technology Co., Ltd. (Shanghai, China). Unless otherwise stated, cultures were incubated under the optimized conditions described below to obtain sufficient and reproducible conidial material for spectrophotometric calibration and validation.

### 2.3. Morphological and Molecular Identification

Morphological identifications were conducted by using an optical microscope (Tianjin Zhenzhen Electronic Technology Co., Ltd., Tianjin, China) to observe colony morphology, hyphal structure, and conidia micro-morphology on PPDA plates. Molecular characterization entailed the extraction of genomic DNA from mycelia and conidia, followed by amplification of the internal transcribed spacer (ITS) region using primers ITS1F (5′-TCCGTAGGTGAACCTGCGG-3′) and ITS4R (5′-TCCTCCGCTTATTGATATGC-3′). PCR amplification and sequencing of the ITS region were performed according to the standard protocol for fungal identification as outlined by White et al. (1990) [27]. Reference ITS sequences were selected from the NCBI GenBank database to include multiple authenticated *B. bassiana* strains and closely related *Beauveria* species for taxonomic comparison. Additional non-*Beauveria* entomopathogenic fungi were included to provide broader phylogenetic context. Multiple sequence alignment was performed before phylogenetic reconstruction. The phylogenetic tree was constructed using the Neighbor-Joining method in MEGA 12.0, and branch support was assessed using 1000 bootstrap replicates.

### 2.4. Optimization of Culture Medium Components for Consistent Conidia Material

To ensure a sufficient and stable supply of conidia for the development of the quantification method, the culture medium was optimized through a series of single-factor experiments. We evaluated the effects of various carbon sources (malt extract, glucose, sucrose, and starch), nitrogen source combinations (N1: NaNO_3_ + NaNO_3_, N2: yeast extract + yeast extract, N3: NaNO_3_ + yeast extract, N4: NaNO_3_ + peptone, N5: NH_4_Cl + peptone, N6: NH_4_Cl + yeast extract), and trace elements (CoCl_2_, FeCl_3_, FeCl_2_, ZnSO_4_, CuSO_4_, CaCl_2_, MnSO_4_) on the sporulation capacity of the *B. bassiana* isolate. Optimal conidia yields were achieved using a modified liquid medium supplemented with malt extract as the carbon source and a combination of sodium nitrate and yeast extract as the nitrogen source, further enhanced by beneficial trace elements. These standardized cultivation conditions were strictly maintained throughout the study to provide uniform biological material for all subsequent spectrophotometric and hemocytometer-based analyses.

### 2.5. Preparation of Conidia Suspensions

The liquid culture was incubated for up to eight days to optimize sporulation. Conidia entrapped within the mycelial network were released by adding 1.0 mL of culture broth to 1.0 mL of a sterile Tween 80 solution (0.1%, *v*/*v*). This mixture was incubated in a water bath at 30 °C with gentle agitation for 30 min to facilitate the dissociation of conidia from hyphal fragments. Subsequently, the mixture was filtered through sterile cotton to separate free conidia from mycelial debris. The resulting filtrate was centrifuged at 12,000 rpm for 12 min, after which the supernatant was discarded to remove residual medium components that could interfere with optical measurements. The conidia pellet was then resuspended in 1.0 mL of a sterile 0.1% (*w*/*v*) agar–water solution. This suspension medium was used to enhance conidia dispersion and minimize sedimentation during optical density measurements and hemocytometer counts.

### 2.6. Hemocytometer Counting and Spectrophotometric Calibration

Conidia concentrations were determined using a hemocytometer (Shanghai Qiujing Biochemical Reagent Instrument Co., Ltd., Shanghai, China) as the reference standard, following established microbiological counting techniques [28]. Serial dilutions of the conidia suspensions were prepared in sterile 0.1% (*w*/*v*) agar–water solution, which served as both the stabilization medium and the analytical blank. Optical density (OD) values were measured at 500 nm, 530 nm, 560 nm, 600 nm, and 650 nm using a UV–visible spectrophotometer (Shanghai Youke Instrument Co., Ltd., Shanghai, China), with the instrument zeroed against the sterile agar–water solution before each measurement. Finally, a linear regression model was established to correlate the resulting OD values at each wavelength with the corresponding conidia concentrations determined by the hemocytometer.

### 2.7. External Validation of the Regression Model

To evaluate the predictive accuracy and robustness of the established spectrophotometric model, an independent validation (blind test) was performed. Thirteen additional batches of *B. bassiana* conidia suspensions, distinct from those used for the calibration curve, were prepared under the same optimized conditions. These validation samples were adjusted to various densities across the linear range and quantified using both the manual hemocytometer counting (as the reference value) and the spectrophotometric method at 650 nm. The predicted concentrations were calculated using the regression equation, and the Relative Error (RE, %) was determined to assess the deviation between the two methods.

### 2.8. Data Analysis

Coefficient of determination (R^2^) values of each wavelength based on the OD values (x axis) and conidia concentrations (y axis) on a scatter plot (linear regression) were measured using the GraphPad Prism 8.0 (GraphPad Software, San Diego, CA, USA). The linear equation corresponding to the highest R^2^ value of *B. bassiana* was considered as the best conidia counting mathematical formula.

## 3. Results

### 3.1. Identification of the Fungal Isolate

**Figure 1:**
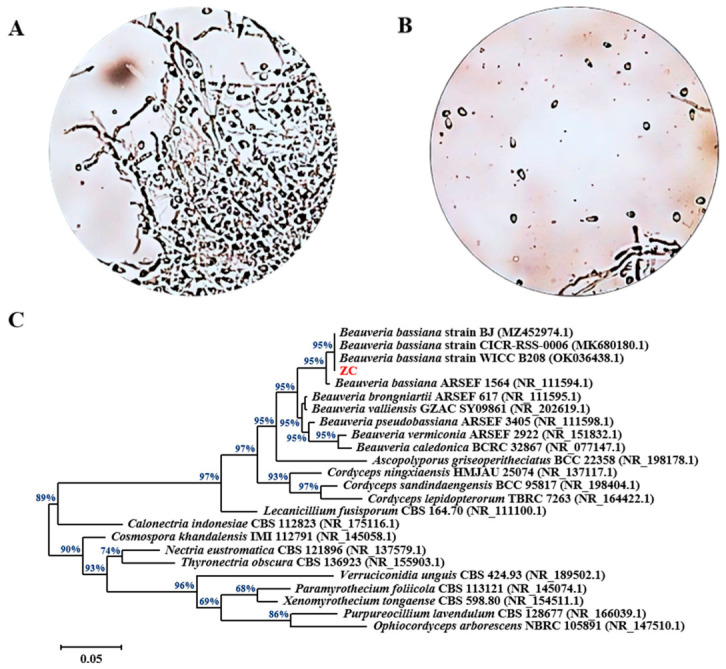
Identification and taxonomic classification of the *B. bassiana* isolate cultured on PPDA medium. (**A**) Microscopic observation of septate hyaline hyphae (magnification: 640×). (**B**) Globose conidia formed on conidiogenous cells (magnification: 640×). (**C**) Phylogenetic tree based on internal transcribed spacer (ITS) rDNA sequences constructed using the Neighbor-Joining method in MEGA 12.0. The numbers at the nodes indicate bootstrap support values based on 1000 replicates. The isolated strain is indicated in red font.

The colonies from cultures derived from Bombyx mori larvae exhibited the typical morphological characteristics of the genus *Beauveria*, as well as several unique microscopic characteristics. The colonies on the PPDA plates were flat and powdery with surface colors ranging from white to light cream. Microscopically, the culture displayed septate, hyaline hyphae (Figure 1A) and globose conidia produced by conidiogenous cells (Figure 1B). The phylogenetic analysis demonstrated that the ITS sequence of the isolate closely aligned with the sequences of the *B. bassiana* type strain deposited at NCBI, thereby further supporting its taxonomic classification (Figure 1C).

### 3.2. Optimization of Conidia Production for Method Establishment

**Figure 2:**
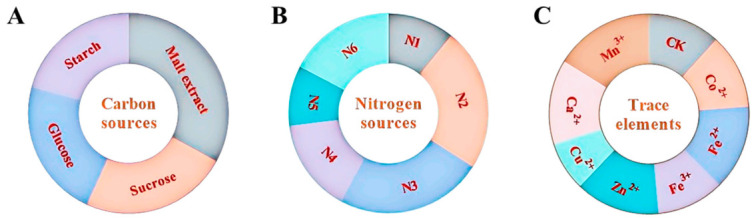
Optimization of cultivation parameters for maximizing *B. bassiana* conidia production in liquid culture medium. (**A**) Carbon source screening (20.0 g/L); (**B**) nitrogen source combinations (2.0 g/L); (**C**) trace elements (7.0 mg/L). Conidia concentrations were assessed after 8 days of cultivation.

Prior to method establishment, cultivation parameters were optimized to obtain sufficient and reproducible conidia samples (Figure 2). The results indicated that malt extract was the most effective carbon source for promoting sporulation. Also, a synergistic effect was observed with the combination of sodium nitrate and yeast extract, which yielded significantly higher conidia concentrations than single-source nitrogen source treatments. Furthermore, most tested trace elements (Co^2+^, Fe^2+^, Zn^2+^, Ca^2+^, and Mn^2+^) enhanced conidia production; however, Fe^3+^ and Cu^2+^ ions exhibited significant negative effects on conidia production. These optimized conditions were utilized to provide a uniform and high-density conidia supply for the subsequent calibration and validation of the spectrophotometric model, rather than as a standalone optimization study.

### 3.3. Evaluation of Conidia Separation and Suspension Efficacy

**Figure 3:**
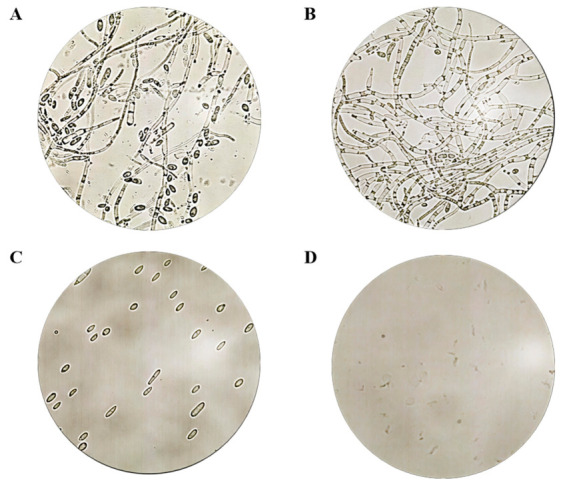
Microscopic evaluation of conidia separation and stabilization of *B. bassiana* suspensions prepared in 0.1% agar–water solution. (**A**) Untreated fermentation broth showing conidia heavily entrapped within dense mycelial structures. (**B**) Suspension on sterile cotton after 0.05% Tween 80 treatment, showing the successful conidia separation. (**C**) Final conidia preparation resuspended in 0.1% (*w*/*v*) agar–water solution, demonstrating uniform dispersion and minimized settling. (**D**) Post-centrifugation supernatant confirming the absence of residual conidia and quantitative sample integrity.

An effectiveness assessment of conidia separation/suspension protocol was performed by microscopic examination (Figure 3). In the untreated fermentation broth, conidia are mainly encapsulated within a dense mycelial network, making it difficult to achieve accurate quantification through direct counting or optical density (OD) measurement (Figure 3A). After treatment with 0.1% Tween 80 and sterile cotton filtration, mycelial fragments were effectively removed, resulting in a suspension mainly composed of free conidia (Figure 3B). Subsequently, highly uniform and well dispersed spore samples were prepared by centrifugation and resuspension in a 0.1% (*w*/*v*) agar–water solution (Figure 3C). The suspension system significantly inhibited the sedimentation of conidia during the measurement period, which is beneficial for the stability of optical detection. Further analysis of the supernatant showed that the centrifugation process did not result in significant loss of conidia, ensuring the quantitative integrity of the sample (Figure 3D). These results demonstrate that the prepared suspensions were highly suitable for subsequent linear regression analysis.

### 3.4. Linear Relationship Between Optical Density and Conidia Concentration

**Figure 4:**
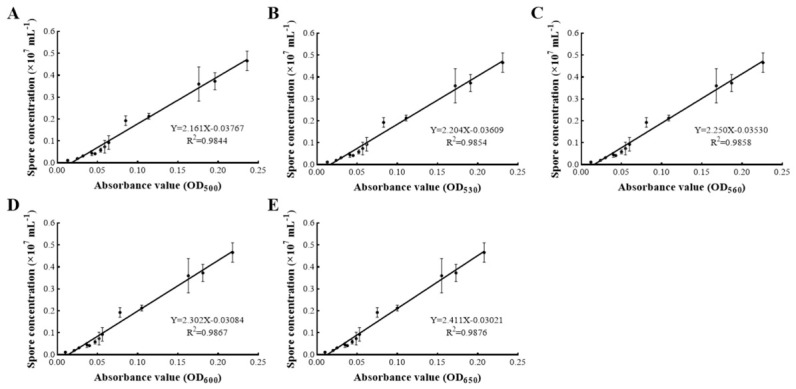
Linear regression models correlating *B. bassiana* conidia concentration with optical density (OD) measured at different wavelengths. (**A**) 500 nm, (**B**) 530 nm, (**C**) 560 nm, (**D**) 600 nm, (**E**) 650 nm. Data are presented as mean ± standard deviation, and vertical bars indicate standard deviation.

The conidia suspension was subjected to serial dilutions, and was measured by hemocytometer counts and UV–visible spectrophotometry, respectively (Figure 4). The optical densities at the five measured wavelengths, including 500 nm, 530 nm, 560 nm, 600 nm, and 650 nm, exhibited a linear correlation to the concentration of conidia. As the concentration of conidia increased, the optical density also increased linearly. According to linear regression analysis, a strong correlation was observed between OD_650_ and conidia concentration (R^2^ = 0.9876). The correlation was determined to be as follows: conidia concentration (×10^7^ mL^−1^) = 2.411 × OD_650_—0.03021 (Figure 4). The regression curves at wavelengths of 500 nm, 530 nm, 560 nm and 600 nm also demonstrated good linear relationships with correlation coefficients (R^2^) of 0.9844, 0.9854, 0.9858 and 0.9867, respectively; however, compared to 650 nm, the determination coefficients at these four wavelengths are slightly lower, indicating a decrease in fitting accuracy. Based on a comprehensive comparison of the performance of different wavelengths, we found that using OD_650_ provided the best prediction performance for *B. bassiana* conidia concentration. Therefore, it was selected as the optimal wavelength for subsequent quantitative determination of conidia. Taken together, the above results show that UV–visible spectrophotometry could help rapidly and accurately estimate *B. bassiana* conidia concentration coupled to proper conidia isolation and dispersion protocols.

**Figure 5:**
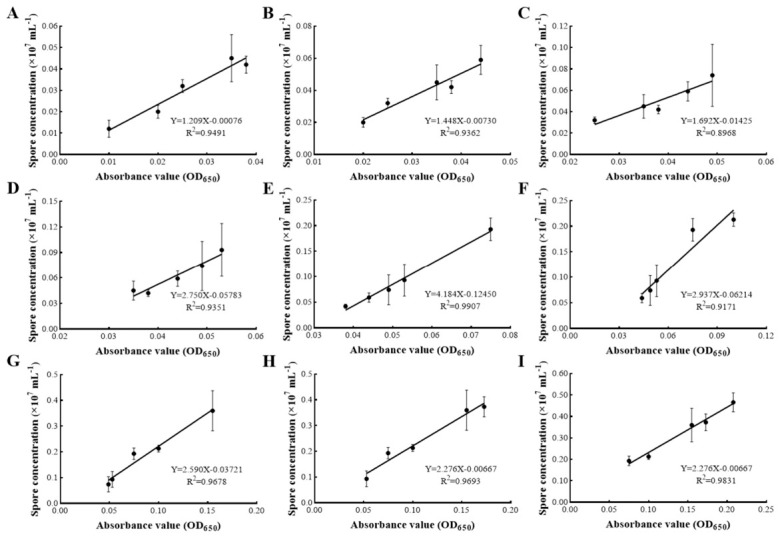
Determination of the optimal linear range for OD_650_-based quantification of *B. bassiana* conidia. Linear regression analysis was performed using sliding windows of five consecutive data points selected from the 13-point OD_650_ dataset. Each subpanel represents one five-point fitting interval arranged according to increasing conidia concentrations. (**A**–**I**) represent nine consecutive five-point fitting intervals arranged from low to high conidial concentration: (**A**) the first five-point interval; (**B**) the second five-point interval; (**C**) the third five-point interval; (**D**) the fourth five-point interval; (**E**) the fifth five-point interval; (**F**) the sixth five-point interval; (**G**) the seventh five-point interval; (**H**) the eighth five-point interval; and (**I**) the ninth five-point interval. The coefficient of determination (R^2^) was compared among intervals to identify the concentration range showing the strongest linearity and thus the most suitable working range for spectrophotometric quantification. Data are presented as mean ± standard deviation, and vertical bars indicate standard deviation.

To further define the most reliable working interval for OD-based quantification, the 13-point dataset obtained at 650 nm was reanalyzed using consecutive five-point subsets (Figure 5). This sliding-window fitting strategy allowed a more detailed evaluation of local linearity across the full concentration range. The highest goodness of fit for the model (R^2^ = 0.9907) was observed at OD_650_ values ranging from 0.038 to 0.075, and the resulting regression equation was as follows: conidia concentration (×10^7^ mL^−1^) = 4.184 × OD_650_ − 0.12450. The results showed that the goodness of fit varied among different five-point intervals, indicating that the linear response of OD_650_ was not equally stable across all tested concentrations. In general, the middle concentration region displayed better linearity than the lowest and highest concentration ranges, where deviation from linearity became more apparent. Based on comparison of the regression coefficients, the interval with the highest R^2^ value was defined as the optimal linear range for OD_650_-based quantification. These results indicate that, although the full 13-point dataset showed an overall strong linear relationship, restricting quantification to the best-performing local interval can further improve the accuracy and robustness of the spectrophotometric method.

### 3.5. Predictive Accuracy and Validation

The reliability of the regression model was confirmed through the analysis of independent validation samples. As shown in Table 1, the conidia concentrations predicted based on the OD_650_ model were consistent with the results obtained from manual hemocytometer counts. Across the tested range, the relative errors were ranging from 13.78% to 18.98%, respectively, which were well within the acceptable threshold for microbial quantitative analysis. This result indicates that the established model has an acceptable agreement and can serve as a reliable alternative to traditional counting methods for the regular evaluation of *B. bassiana*.

## 4. Discussion

Accurate and reproducible quantification of conidia is essential for both fundamental research on fungi and for application purposes such as the use of entomopathogenic fungi against insect pests. In order to meet this demand, an OD-based spectrophotometric calibration protocol was developed to rapidly quantify the number of *B. bassiana* conidia, with its accuracy highly dependent on the ability to create uniform spore suspensions as well as establish species-specific calibration curves [13,16]. The results showed that optical density values measured at visible wavelengths were positively correlated with hemocytometer-based conidial concentrations within appropriate working ranges. Among the wavelengths tested, OD_650_ provided the optimum linear model under the experimental conditions used in this study. Independent validation using separate conidial batches further indicated that the selected OD_650_ model could provide acceptable predictive agreement for routine estimation. Therefore, the present work provides a practical, species-specific calibration protocol for standardized *B. bassiana* conidial quantification in biopesticide quality control and bioassay applications.

When *B. bassiana* is produced in liquid media, the conidia may be entangled with mycelium. In this study, Tween 80 successfully disentangles the conidia from the mycelia, followed by filtration and centrifugation. The spores were then kept suspended in an aqueous 0.1% agar solution for better dispersion and minimal sedimentation. Similar methods with the aim of improving suspension of particles were found to enhance measurement accuracy in the literature [3]. The selection of a suitable wavelength for estimation is an important factor affecting the accuracy of this method. Despite the common use of spectrophotometry to estimate biomass levels, the optical properties of fungal spores vary with wavelength, due to the morphology of shape (size, structure, texture, color) [29]. The results obtained in our experiments showed higher correlation coefficients for the wavelength of 650 nm than that measured with the 500 nm, 530 nm, 560 nm and 600 nm wavelengths, which indicates that OD_650_ measurements are more sensitive to variations within this range of concentrations. Similar observations for wavelength-specific absorbance have been found when determining concentrations of conidia suspensions of *Trichoderma* and *Cladosporium fungi*. Waghunde et al. used optical density as a rapid tool for estimating the conidial count of *Trichoderma viride* and showed that absorbance measurements could be correlated with conidial concentration within a defined working range [15]. Oghaz et al. further adjusted and quantified UV–visible spectrophotometric analysis for *Cladosporium* spp. conidia in water suspension and demonstrated that reliable prediction requires the establishment of an appropriate wavelength and a fungus-specific calibration curve [17]. Therefore, the OD_650_ model developed in the present study should be considered a *B. bassiana*-specific calibration protocol under the agar–water suspension conditions used here.

Previous studies have demonstrated that using UV–visible spectrometry for quantification of spores from various fungi is a promising method, including *Fusarium*, *Penicillium*, *Trichoderma*, *Aspergillus*, and *Cladosporium* [13,15,16,17,29]. These studies demonstrated a strong linear correlation between optical density and conidia concentration within the tested range, thereby reinforcing the extensive applicability of spectrophotometry for quantifying fungal conidia. Compared to conventional counting using a hemocytometer, the optical spectrometry technique has several advantages including in particular reduced process times, increased throughput of samples to be processed, and lower operator-to-operator variance [30]. Consequently, this technique is particularly well-suited for routine analytical applications, such as monitoring fermentation processes, ensuring the quality of conidia preparations, and conducting standardized bioassays of *B. bassiana*. Notably, this method is user-friendly, requires no additional instrumentation or complex pre-processing, and can be seamlessly integrated into conventional fermentation processes and offline quality control programs.

The morphological variation, pigmentation, and aggregation state of conidia have been demonstrated to significantly influence their optical properties, thereby affecting the accuracy and robustness of spectrophotometric calibration models [16,29]. Schütz et al. (2020) specifically highlighted that morphological differences among various *Trichoderma* conidia can markedly alter their photoresponsive behavior, demonstrating the importance of developing species-specific calibration models [16]. Similar observations have been reported in studies of *Cladosporium*, where the calibration curve is essential for accurately predicting conidia concentrations [17]. In alignment with these findings, the regression model developed in this study is specifically calibrated for *B. bassiana* under the described experimental conditions and cannot be directly applied to other fungal species without further validation. It is important to note that species-specific calibration is a common requirement in industrial biological process monitoring and does not compromise the practicality of this methodological framework in real-world applications.

Furthermore, a good linearity observed in this study at 650 nm is also consistent with those reported by several fungi under optimal detecting wavelengths. Thus, UV–visible spectroscopy could be used to quickly estimate conidial concentration, as long as adequate calibration and sample preparation are carried out [13,15]. Unlike previous investigations that primarily relied on the coefficient of determination (R^2^), this study assessed the practicality of the model by independent validation tests. The low relative error observed in the blind sample test suggests that a 0.1% (*w*/*v*) agar–water solution not only ensures the uniform dispersion of conidia but also provides a stable optical measurement environment. This result confirms that the obtained calibration model did not over-fit on the original data, and can be considered appropriate regarding the requirements of high-quality studies conducted in laboratories. Previous work has already demonstrated the applicability of optical approaches to spore estimation in entomopathogenic fungi [26]. Building on this foundation, the present study provides a narrower but more operationally focused protocol for *B. bassiana*, emphasizing suspension stabilization, OD_650_-based calibration, working-range definition, and independent validation using separate conidial batches. Therefore, the contribution of the present work lies not in the first demonstration of spectrophotometry for *B. bassiana*, but in providing a defined suspension system, wavelength-specific calibration, working-range selection, and validation framework for routine *B. bassiana* quality control and bioassay applications.

In addition to being a simple and easy-to-use method in the laboratories, the spectrophotometric quantitative framework developed in this study exhibits significant potential for the industrial-scale production of *B. bassiana*. In large-scale liquid fermentation processes, real-time monitoring of conidia concentration is crucial for determining the optimal harvest time; however, traditional methods often fall short in terms of rapidity and stability. The 0.1% (*w*/*v*) agar–water suspension system employed in this study effectively mitigates optical interference caused by conidia sedimentation, thereby providing stable measurement conditions suitable for integration into online sensing systems. In comparison to conventional manual counting techniques, the standardized regression model based on a wavelength of 650 nm offers objective and reproducible evaluation criteria for quality control across different production batches, and future work could focus on the applicability of such models in real-life applications. From an agricultural standpoint, the proposed spectrophotometric method enhances the consistency in the preparation of *B. bassiana* inocula, thereby mitigating dosage-related variability in laboratory and greenhouse bioassays, as well as in field evaluations. The improved standardization of conidial concentrations is anticipated to augment the reproducibility of pest control efficacy assessments and facilitate more reliable comparisons among biological control studies. Consequently, this approach may contribute to a more rigorous evaluation and practical application of entomopathogenic fungi within integrated pest management programs.

## 5. Conclusions

This study established an agar–water-assisted OD_650_ calibration protocol to estimate conidial concentrations in *B. bassiana*. A 0.1% (*w*/*v*) agar–water suspension was used to reduce conidial aggregation and sedimentation and to maintain a stable dispersion during optical measurement. A species-specific calibration model was developed at 650 nm and independent validation using separate conidial batches showed acceptable predictive agreement with hemocytometer-based counts. The method is simple to perform, requires short analysis time, and produces results consistent with traditional microscopic counting. Overall, this study provides a practical means for standardizing conidial dosage in agricultural biopesticide research and application, thereby supporting more consistent evaluation and deployment of entomopathogenic fungi in pest management.

## Figures and Tables

**Table 1:** External validation of the OD_650_ spectrophotometric model using independent *B. bassiana* conidia batches.

Sample No	Hemocytometer Count (×10^7^ mL^−1^)	OD_650_ Value	Predicted Concentration (×10^7^ mL^−1^)	Relative Error * (%)
01	0.0425	0.038	0.0345	18.84
02	0.0450	0.039	0.0387	14.05
03	0.0475	0.039	0.0387	18.58
04	0.0575	0.041	0.0470	18.18
05	0.0950	0.049	0.0805	15.25
06	0.1150	0.053	0.0973	15.43
07	0.1225	0.055	0.1056	13.78
08	0.1450	0.058	0.1182	18.50
09	0.1750	0.064	0.1433	18.13
10	0.1975	0.068	0.1600	18.98
11	0.2075	0.071	0.1726	16.84
12	0.2200	0.074	0.1851	15.86
13	0.2575	0.080	0.2102	18.36

* Relative Error (%) denotes the deviation between the two methods across the linear range.

## Data Availability

All relevant data are within the manuscript, and the data are available from the corresponding author on reasonable request.

## References

[1] Sharma A., Sharma S., Yadav P.K. (2023). Entomopathogenic fungi and their relevance in sustainable agriculture: A review. Cogent Food Agric..

[2] Rajput M., Sajid M.S., Rajput N.S., George D.R., Usman M., Zeeshan M., Iqbal O., Bhutto B., Atiq M., Rizwan H.M. (2024). Entomopathogenic Fungi as Alternatives to Chemical Acaricides: Challenges, Opportunities and Prospects for Sustainable Tick Control. Insects.

[3] Papantzikos V., Mantzoukas S., Eliopoulos P.A., Servis D., Bitivanos S., Patakioutas G. (2024). Evaluation of various inoculation methods on the effect of *Beauveria bassiana* on plant growth of kiwi and on *Halyomorpha halys* infestation: A two-year field study. Biology.

[4] Sain S.K., Kranthi S., Kranthi K.R., Monga D., Paul D., Prasad Y.G. (2024). Diversity study of *Beauveria bassiana* for identifying virulent strains to manage *Bemisia tabaci* in cotton. Appl. Microbiol. Biotechnol..

[5] Iida Y., Higashiet Y., Nishi O., Kouda M., Maeda K., Yoshida K., Asano S., Kawakami T., Nakajima K., Kuroda K. (2023). Entomopathogenic fungus *Beauveria bassiana*–based bioinsecticide suppresses severity of powdery mildews of vegetables by inducing the plant defense responses. Front. Plant Sci..

[6] Bitencourt R.O.B., Queiroz R.R.S., Ribeiro A., Ribeiro Y.R.d.S., Boechat M.S.B., Carolino A.T., Santa-Catarina C., Samuels R.I. (2024). Encapsulation of *Beauveria bassiana* conidia as a new strategy for the biological control of *Aedes aegypti* larvae. Sci. Rep..

[7] Mascarin G.M., Jaronski S. (2016). The production and uses of *Beauveria bassiana* as a microbial insecticide. World J. Microbiol. Biotechnol..

[8] Fenibo E.O., Matambo T. (2025). Biopesticides for sustainable agriculture: Feasible options for adopting cost-effective strategies. Front. Sustain. Food Syst..

[9] Mawcha K.T., Malinga L., Muir D., Ge J., Ndolo D. (2025). Recent advances in biopesticide research and development with a focus on microbials. F1000Research.

[10] Vega F.E., Goettel M.S., Blackwell M., Chandler D., Jackson M.A., Keller S., Koike M., Maniania N.K., Monzón A., Ownley B.H. (2009). Fungal entomopathogens: New insights on their ecology. Fungal Ecol..

[11] Zimmermann G. (2007). Review on safety of the entomopathogenic fungi *Beauveria bassiana* and *Beauveria brongniartii*. Biocontrol Sci. Technol..

[12] OECD (2025). Consensus Document on Beauveria bassiana Strains as Microbial Plant Protection Products: Regulatory Considerations.

[13] Caligiore-Gei F.P., Valdez G.F. (2015). Adjustment of a rapid method for quantification of *Fusarium* spp. conidia suspensions in plant pathology. Rev. Argent. Microbiol..

[14] Valdez J.G., Piccolo R.J. (2006). Use of spectrophotometry as a tool to quantify the sporulation of *Penicillium allii* in garlic lesions. Fitopatol. Bras..

[15] Waghunde R., John P., Naik B., Solanky K., Sabalpara A. (2010). Optical density: A tool for the estimation of conidia count of *Trichoderma viride*. J. Biopestic..

[16] Schütz G., Haltrich D., Atanasova L. (2020). Influence of conidia morphology on spectrophotometric quantification of *Trichoderma* inocula. BioTechniques.

[17] Oghaz N.A., Hatamzadeh S., Rahnama K., Moghaddam M.K., Vaziee S., Tazik Z. (2022). Adjustment and quantification of UV–visible spectrophotometry analysis: An accurate and rapid method for estimating *Cladosporium* spp. conidia concentration in a water suspension. World J. Microbiol. Biotechnol..

[18] Vanhauteghem D., Demeyere K., Callaert N., Boelaert A., Haesaert G., Audenaert K., Meyer E. (2017). Flow cytometry is a powerful tool for assessment of the viability of fungal conidia in metalworking fluids. Appl. Environ. Microbiol..

[19] Hao B., Chen S., Qiu W., Liu K., Domenech A.C., Fernandez J.A.B., Shen J., Li M., Yang X. (2026). High-throughput quantitative detection of *Pseudoperonospora cubensis* sporangia in cucumber by flow cytometry: A tool for early disease diagnosis. Agronomy.

[20] Li C., Ma X., Deng J., Li J., Liu Y., Zhu X., Liu J., Zhang P. (2021). Machine learning-based automated fungal cell counting under a complicated background with ilastik and ImageJ. Eng. Life Sci..

[21] Ren Z., Liang K., Zhang Y., Song J., Wu X., Zhang C., Mei X., Zhang Y., Liu X. (2025). An intelligent method for detection of small target fungal wheat conidia based on an improved YOLOv5 with microscopic images. Plant Methods.

[22] Kulshreshtha A.K., Singh O.N., Wall G.M. (2010). Pharmaceutical Suspensions.

[23] Petrikkou E., Rodríguez-Tudela J.L., Cuenca-Estrella M., GómEz A., Molleja A., Mellado E. (2001). Inoculum standardization for antifungal susceptibility testing of filamentous fungi pathogenic for humans. J. Clin. Microbiol..

[24] Rodríguez-Tudela J.L., Cuenca-Estrella M., Díaz-Guerra T.M., Mellado E. (2001). Standardization of antifungal susceptibility variables for a semiautomated methodology. J. Clin. Microbiol..

[25] Pandey N., Jain R., Pandey A., Tamta S. (2018). Optimisation and characterisation of the orange pigment produced by a cold-adapted strain of *Penicillium* sp. (GBPI_P155) isolated from a mountain ecosystem. Mycology.

[26] Mora-Pérez C.J., Favela-Torres E., Montesinos-Matias R., Berlanga-Padilla A.M., Méndez-González F. (2025). Colorimetry has practical advantages over spectrophotometry for estimating entomopathogenic fungi spore concentration in aqueous suspensions. Folia Microbiol..

[27] White T.J., Bruns T., Lee S., Taylor J., Innis M.A., Gelfand D.H., Sninsky J.J., White T.J. (1990). Amplification and direct sequencing of fungal ribosomal RNA genes for phylogenetics. PCR Protocols: A Guide to Methods and Applications.

[28] Harley J.P., Prescott L.M. (2002). Laboratory Exercises in Microbiology.

[29] Kulik M.M., Johnson R.M. (1969). Feasibility of characterizing conidia color in *Aspergillus* spp. by reflectance spectrophotometry. Mycologia.

[30] Stevenson K., McVey A.F., Clark I.B.N., Swain P.S., Pilizota T. (2016). General calibration of microbial growth in microplate readers. Sci. Rep..

